# Integration in Medicine-Pediatrics Residency Programs: A Mixed-Methods Study

**DOI:** 10.7759/cureus.113288

**Published:** 2026-07-24

**Authors:** Rose Barham, Staci R Eisenberg, Meaghan N Roszyk, Claire J Volpe, Daniel Wells, Benjamin Doolittle

**Affiliations:** 1 Internal Medicine-Pediatrics, Geisinger Health System, Danville, USA; 2 Internal Medicine-Pediatrics, Brigham and Women's Hospital, Harvard Medical School, Boston, USA; 3 Primary Care, Christiana Care Health System, Wilmington, USA; 4 Yale Program for Medicine, Spirituality, and Religion, Yale School of Medicine, New Haven, USA; 5 General Pediatrics, The University of Tennessee Health Science Center College of Medicine, Memphis, USA; 6 Internal Medicine-Pediatrics, Yale School of Medicine, New Haven, USA

**Keywords:** combined training, graduate medical education, inclusion, med-peds, professional integration, residency

## Abstract

Background

There are a variety of combined residency training programs, including an increasing number of medicine-pediatrics (Med-Peds) residency programs. Successful integration of these residents into the learning environments of both departments is important to train competent, successful physicians. Little is known about the factors that contribute to successful integration.

Objective

To assess Med-Peds integration and explore factors essential for the successful integration of combined residents into their host departments.

Methods

To further elaborate on findings from a national survey, a convenience sample of 33 Med-Peds program leaders participated in focus groups of 3-5 participants to explore factors associated with successful integration of Med-Peds programs into their host departments. Data were collected in March 2025 during the national meeting of the Med-Peds Program Directors Association (MPPDA). Focus group transcripts were analyzed using inductive qualitative analysis.

Results

One hundred percent of programs that participated in focus groups (33/33) were either “somewhat well-integrated” or “well-integrated” into their medicine department, and 30/33 (91%) were “somewhat well-integrated” or “well-integrated” into their pediatrics department. There was no clear statistical difference between the medicine and pediatrics departments (p = 0.054). The focus groups identified four factors associated with successful integration of Med-Peds programs: residents’ involvement in cross-program activities, the development of personal relationships across departments, the program’s administrative inclusion, and its cultivation of programmatic identity.

Conclusions

Med-Peds programs are well integrated into their host departments, according to program leaders. Fostering these qualities of integration is critical for successful combined programs.

## Introduction

According to the Accreditation Council for Graduate Medical Education, there are 79 medicine-pediatrics (Med-Peds) residency programs, offering 398 intern positions [1]. While this is the largest cohort of dual-training programs, there are 14 other dual-training programs, such as internal medicine and emergency medicine, and child psychiatry and pediatrics. Across all combined residency programs, including Med-Peds, 578 residents match annually [1].

The Med-Peds programs were independently accredited by the ACGME in 2006 [2]. While this prompted an increased allocation of resources and program support specifically to Med-Peds programs [2], data about successful Med-Peds integration are sparse. In 2006, a national survey of Med-Peds program graduates showed that they felt equally well prepared in medicine and pediatrics (80% vs. 77%, respectively; p = 0.305) [3]. A five-year cumulative national survey by Chamberlain JK et al. among Med-Peds graduates demonstrated overall satisfaction with their training, with slightly higher satisfaction with medicine training compared with pediatrics training (86% vs. 83% satisfaction; p < 0.01) [4].

There has been some exploration of the trainee experience during residency in other combined programs. In one study of combined pediatrics-anesthesiology residents, most residents had a higher sense of belonging and self-identification in anesthesiology than in pediatrics [5]. A recent national survey comparing residency training satisfaction between categorical dermatology and combined internal medicine-dermatology (Med-Derm) trained physicians found higher satisfaction among categorical dermatology residents, noting that 36% of Med-Derm graduates believed they were treated differently from both their internal medicine and dermatology colleagues [6].

For dual-program residents, successful integration into the culture and pedagogic milieu of their host departments is necessary for their academic progress. Integration involves residents rotating through host rotations and programs in a way that allows them to function well and have adequate support for their roles. While the importance of a supportive learning environment and a resident’s sense of belonging is well documented [7,8], few studies explore the specific concept of integration of combined residency programs. Moreover, most literature examining combined programs has focused on practice patterns and career satisfaction after training rather than the training experience itself. Since 2012, to our knowledge, there has not been a formal assessment of Med-Peds program integration. To understand the scale of the integration challenge and identify targets for program improvement, we sought survey data and qualitative exploration of factors essential for successful integration.

## Materials and methods

Study design 

Additional questions were added to the program directors’ national survey, and focus groups were conducted to understand the driving forces behind the survey results. A convenience sample of Med-Peds residency program leaders was invited to participate in focus groups to explore themes related to the integration of Med-Peds programs. These focus groups were conducted during the Med-Peds Program Directors Association (MPPDA) annual meeting, held from March 26 to 28, 2025, where all MPPDA participants were invited to participate in the focus groups through announcements and electronic communication. The focus groups took place in the conference center during breaks in the program directors’ meeting. Participation was voluntary. Informed consent was obtained. The Yale University Institutional Review Board approved this study (IRB: 2000033160). The Executive Committee of the MPPDA gave permission for this study to be conducted during the meeting.

Data collection 

Following the administration of the national survey, program leaders in a convenience sample were interviewed by three investigators in focus groups comprising 3-5 participants. Basic demographic information, such as age, gender, ethnicity, and length of career, was collected. A brief survey was conducted to quantify the perception of program integration. The focus group guide was developed by the research team to explore what integration means and what facilitates it. In the focus group discussions moderated by the study investigators, a standardized list of open-ended questions was used to explore themes of integration of Med-Peds programs, with necessary follow-up questions to explore emergent ideas (Appendix 1). The interviews were recorded and transcribed. All identifying data were removed from the transcription to preserve anonymity.

Data analysis 

The investigators reviewed the transcripts independently and utilized line-by-line coding to develop an initial codebook. Following initial discussion, all transcripts were coded by at least two members of the research team. Three members of the team met to identify themes from coding using a grounded theory-based approach to identify naturally occurring themes based on the lived experiences of the program leaders [9]. Discrepancies were reconciled through a deliberative modified delphi approach [10] where the research team met to discuss agreement on code application.  

JMP software was used to perform the Mann-Whitney U test to compare the responses of integration between the medicine and pediatrics departments [11] from the survey data.

## Results

Participants

**Figure 1:**
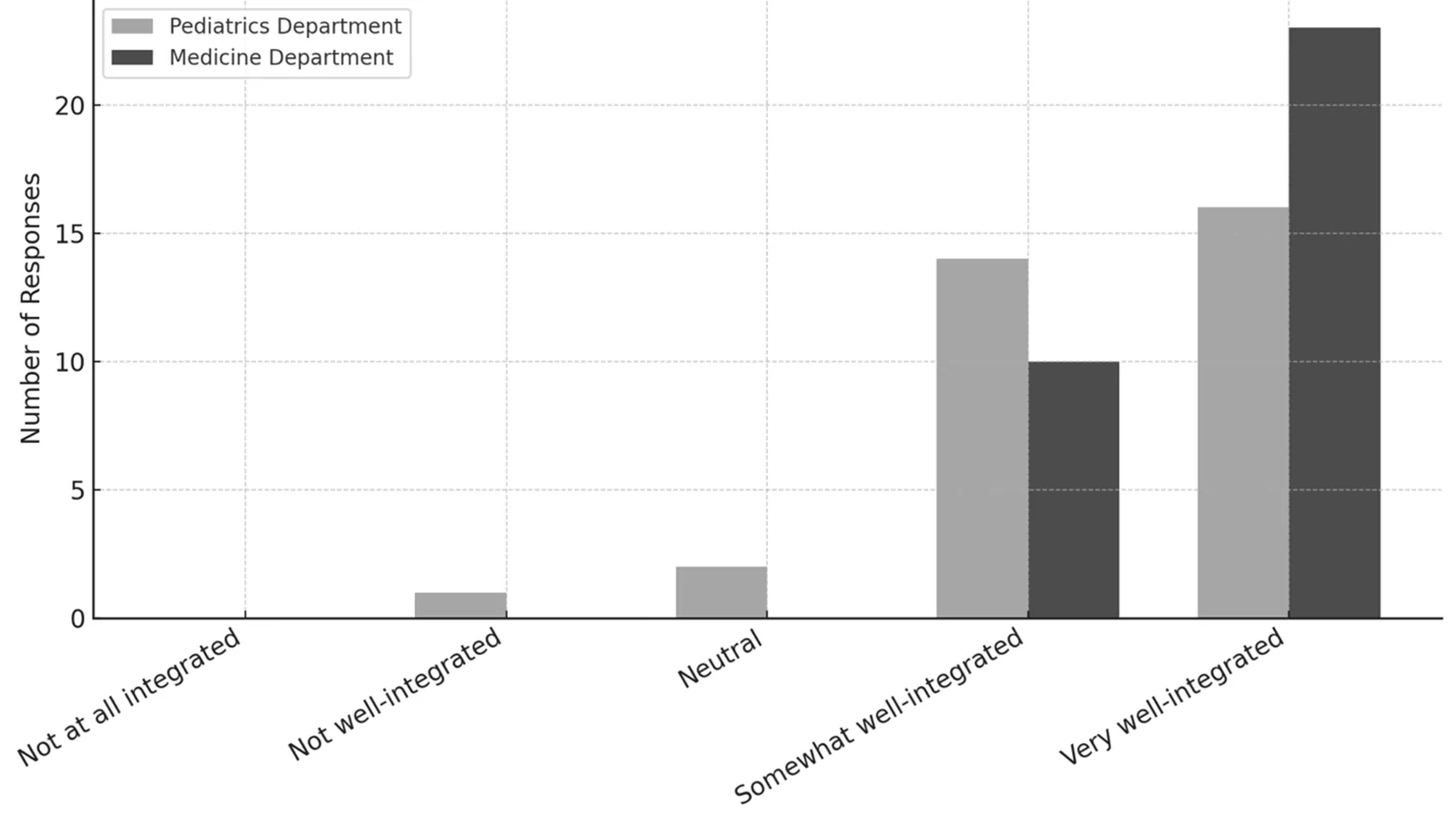
Integration of Med-Peds residency programs by department.

Thirty-three program leaders, from nine focus groups, completed interviews representing 33/79 (42%) of programs (Table 1). Participants stated that their programs were “well-integrated” into their host departments. All programs (33/33, 100%) were either “somewhat well-integrated” or “well-integrated” into their medicine department, and 30/33 (91%) were “somewhat well-integrated” or “well-integrated” into their pediatrics department (Figure 1).

**Table 1:** Demographic characteristics of participants (n = 33).

Characteristic	Value
Age, median (range)	44 (31-62)
Gender	
Male	10 (30%)
Female	23 (70%)
Ethnicity	
White	23 (70%)
Hispanic	2 (6%)
Black	2 (6%)
Asian	1 (3%)
No answer	5 (15%)
Years in program leadership, median (range)	10 (1-27)
Program setting	
Academic medical center	29 (88%)
Community medical center	2 (6%)
No answer	2 (6%)

There was no statistically significant difference between departments (Mann-Whitney U statistic = 414.0, p = 0.054).

Thematic analysis

**Figure 2:**
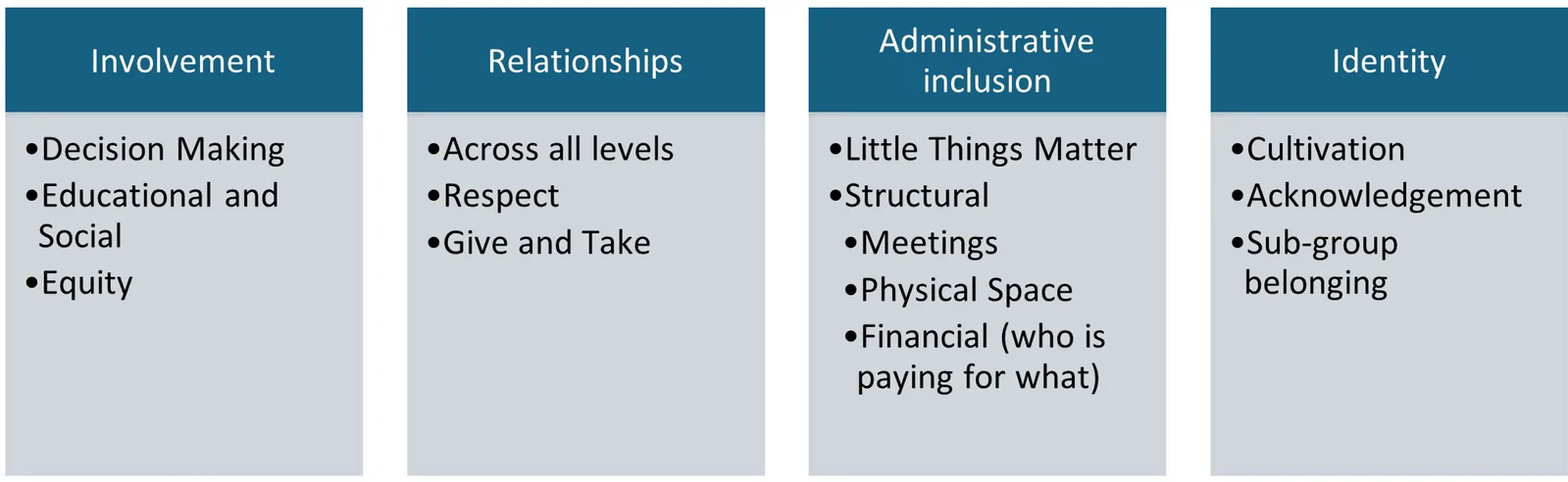
Major themes of successful integration of medicine-pediatrics programs.

Four major themes were identified: resident involvement across departments, interdepartmental relationships, administrative inclusion, and collective program identity (Figure 2). Resident involvement was highly dependent on a range of respectful, personal relationships within the program, as well as the structure of the residency programs, where meetings and physical spaces increased face time between residents. While there is some tension in cultivating a unique Med-Peds identity and maintaining a sense of belonging to a categorical program, this tension did not inherently challenge program integration.

Resident and leadership involvement

Residents’ involvement in cross-program activities, including clinical care, academic work and improvement, and social activities, was essential to good integration.

“From an administrative standpoint, [it is important] that their opinions and their ideas are integrated.”

“[They] can access kind of support and resources in a very similar way to their categorical colleagues, that they feel like they are not necessarily identified as an outsider when they're working in their categorical settings, and that they feel like there's adequate support.”

“When our residents are highly considered for chief positions, that's a good sign of being well integrated and well regarded.”

Involvement in decision-making repeatedly emerged as a key component in supporting integration. When the combined programs are not included in a categorical residency’s decision-making, they feel forgotten or undervalued. Similarly, exclusion of a combined program’s leadership from discussions can lead to logistical problems.

“If we weren't included in the planning stage, our residents would be completely eliminated from having the opportunity to attend.…just being in that planning phase of some of these extras is really helpful… that really helps us get our residents to these other opportunities that categorical residents have.”

Here, equity emerged as a critical aspect of involvement:

“[An] equitable seat at the table when it comes to looking at the clinical learning environment, creating educational opportunities, research opportunities.”

“I think about resident treatment as equals to their categorical peers. Are their experiences equal?”

Equity also encompassed a sense of shared responsibility.

“The things that make it successful is that it's scratching each other's backs, right…the number of times we're able to do things to help support the categorical programs, it shows that we're not just a needy bunch who's exceptional in all these different ways, but we really do want to make things as equitable as possible. We get a lot of goodwill when they see that goodwill.”

Interdepartmental relationships

Building personal relationships across departments mattered in clinical care, meetings, scholarly collaboration, work-based social events such as “retreats,” and social connections.

**Figure 3:**
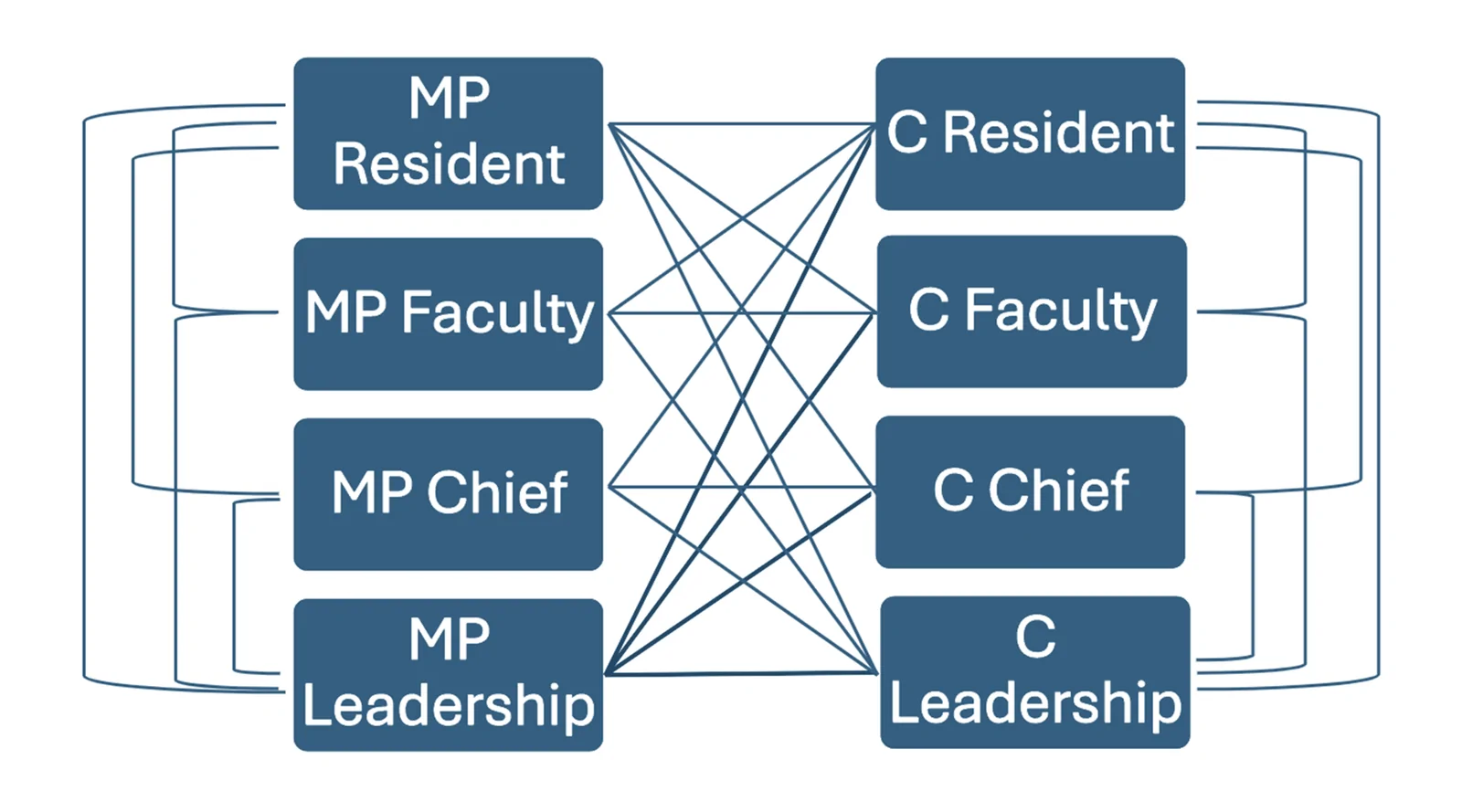
Relationship diagram of Med-Peds stakeholders and department leaders. MP: Med-Peds; C: Categorical (internal medicine or pediatrics).

Relationships were identified as important for improving all aspects of integration. These relationships were more effective when they incorporated a variety of roles and levels within a program (Figure 3).

“Not only are [we] mentoring our own residents in Med-Peds, but we're seen as people who can also mentor pediatric residents and seen as people who can mentor medicine residents.”

“They tap Med-Peds faculty for information specific to the categoricals. So, not this sense [that] your input is only desired if we are discussing Med-Peds residents, but ‘he has expertise in refugee health.’ He’s who we are going to draw on in the medicine program and the peds program.”

The critical factor in those relationships was mutual respect.

“I do think that's possible to work alongside peds and work alongside medicine. But then have people appreciate the extra thing you add or the thing that you don't do as much that they're happy to show you.”

“It is also the language that's used. Are you considered a part of their residency, or are you, as I was told by a previous program director, the ‘ugly stepchild, and you will do what we say?’”

Participants were cognizant of contributing to program integration to ensure that the Med-Peds program was putting effort into the relationship “so that we're seen as contributors, not challenges.”

“Our residents are part of the conversations and are asked to be part of the solutions whenever issues arise in the residency programs.”

While relationships at every level were important, the strength of relationships at the leadership level was particularly impactful:

“How well integrated is the Med-Peds program director with the categorical program directors? If there's friction there, it’ll go all the way down [the program].”

“I will say one of my chairs had worked with Med-Peds previously and is incredibly supportive. The other one still does not get Med-Peds, and that's why, on that side, I don't feel as supported.”

Administrative inclusion**

Being remembered by categorical programs was highly impactful for combined program residents, even for things that were acknowledged as relatively low importance. This inclusion was distinct from respect because participants often recognized that mistakes and lapses were unintentional but still impactful.

“The little things are actually important for them. If the Peds program gets the T-shirts handed out, they need to get a T-shirt.” Or, “We didn’t buy enough sandwiches.”

“Every year we have to remind people to keep our PGY-4s on the email list, so they're not dropped off like all the other PGY-3s.”

“When medicine sends out [the match list], internal medicine puts their name and my name as PDs, and so it comes from all of us. On the Peds side, the list gets sent out with Peds in a list from the Peds program directors. I don't think it’s meant to be a slight or exclusionary, but it is that example of ‘here, I am more well included’ and ‘here, I am less well included.’”

There were several structural components that promoted inclusion. These structural components included factors that increased “face time” through physical proximity and regular meetings. Many programs that were “well-integrated” had regular combined meetings.

“The more face time you have with your categorical leadership, then they're like, ‘Oh yeah, these seven meetings I was having this week were about this new thing we're developing. I didn't ask you yet. How does that work for your residents?’”

“Every time there's a scheduled meeting, we meet. We're not going to skip meetings, because if you don't meet, then you're going to miss something.”

Physical space contributed significantly to face time by increasing the likelihood of unplanned interactions, such as side conversations that facilitate innovation. Multiple participants described advocating for these shared spaces to increase contact.

“Because the whole medicine suite is the same…there's no way I could be as integrated with Peds.”

“I just sit on the other side [of the suite] once a week and just sit touch down there…that's what our chief does too.”

Allocation of resources was another structural aspect of inclusion that played a notable role in feeling integrated:

“[If there is] confusion over where ownership or responsibility lies, ‘this is medicine, we pay’ or ‘this person's employed through medicine; therefore, this is not my responsibility.’ Those sorts of things sometimes get in the way. Especially when founding members at any level are no longer at the table. It’s questions like, ‘How did we come up with this? Who owns the clinic? Who's owning your health benefits? Who's owning your program funding?’ That is very tricky...to make you still feel welcome and supported when you're like, okay, we need these things. They're very important things, but they're also big budget items.”

“I agree about the budget piece. I have to defend, not infrequently, the ‘double dipping’ of resources like graduation or other retreats. I have to continue to remind people that we also, our PGY3s, cover so that all the graduates can be at graduation, which means their colleagues, their friends who they were in the trenches with, they don't get to see [them] graduate. And that's an L for us, but we do it. That's one of the benefits we're able to provide.”

Cultivating programmatic identity

Program identity stressed the importance of appreciating the variety of residency identities, which were not inherently in competition with program integration.

“Isn't the goal to appreciate what everybody brings to the table? I want them to be so proud of their own identity and their own group. I don't think that means that they're saying that they're better than me. I would hope that we could do the same thing and have that really strong identity and not be like ‘we're better, or you're not,’ but just ‘we have a strong identity, you have the strong identity of this other thing. We can learn from each other.’”

“The identity comes from the camaraderie. The identity comes from the relations of the people who are Med-Peds.”

“We've been pretty intentional this year about thinking about our Med-Peds identity, because…it wasn’t feeling as distinct as the categorical medicine and peds. So, I think talking to the residents, like, who are we? What do we value as a Med-Peds residency program? And of course, still being fully medicine and fully peds, also having that kind of carved-out space…we can write it down, we can talk about it; it's very explicit.”

Also important was the combined program’s recognition of its shared identity across two programs.

“When I did my first switch to my pediatric side, I also bumped into the program director. She also knew my full name. I didn't know that she knew I existed. But I think that integration of saying, ‘you're one of our own,’ was a very clear message I got early in intern year. I've heard that from residents, too, that when the categorical leaders know them by name, or something personal about them, it really makes them feel welcome.”

A component of this program identity was subgroup identity. For instance, if there was an urban health track or a global health track in which both Med-Peds and categorical residents participated, that shared identity helped program integration.

“I think our residents sometimes feel they're more integrated into our medicine program because the function of the medicine program puts them in smaller teams where, every time they go to medicine, they're on that team. I think that has facilitated more integration than peds, which doesn't have a similar function.”

However, tension between individual and group identity can arise when there is a lack of appreciation for different identity groups.

“I'm very cognizant that one of the greatest strengths of my program is that we have a strong Med-Peds identity. But one of the greatest weaknesses is that we have a very strong Med-Peds identity, and this has led to individuals on the categorical side either viewing us as elitists or alarmists, and this has changed the way that I approach the categorical program directors when we need change or when I feel there are problems.”

“The way we come off can seem unappreciative because we have that comparison. So, I think it's easier to, perhaps, slight one side over the other because sometimes strong identity feels that it's at the expense of others.”

Still, the consensus was that multiple program identities do not affect the integration of the programs.

“I think the other thing [that] is important about Med-Peds integration is that the residents understand the duality of that relationship, that being integrated doesn't mean that your skill set is also not appreciated as a separate entity. That duality is very difficult to balance, because you want to be integrated. But you don't want to lose that individuality and the uniqueness that we bring to the table.

I think it stems from being seen for their individual career path…They want to be seen; that's an individual path that's very clearly different. But you still want to be part of that.”

## Discussion

This project explores the factors that Med-Peds program leaders identify as crucial for successfully integrated Med-Peds programs. The description of good program integration was explored in the focus groups. The focus groups identified four factors associated with successful integration of Med-Peds programs: residents’ involvement in cross-program activities, the development of personal relationships across departments, the program’s administrative inclusion, and its cultivation of programmatic identity. The key findings echo Ferman’s definition of inclusion in the workplace: a place where “people of all identities and many styles can be fully themselves while also contributing to the larger collective, as valued and full members” [12]. While Med-Peds programs are generally well integrated according to the survey data, the qualitative findings suggest that a commitment to inclusion and parity is beneficial.

Shore LM et al. further expanded the definition of inclusion into a framework that includes key principles of inclusion: feeling safe, being involved in the workgroup, feeling respected and valued, influencing decision-making, supporting authenticity, and recognizing, honoring, and advancing diversity [13]. We found four parallel themes. Resident and leadership involvement in the workgroup occurred through shared information, resources, collaboration, and decision-making. We found that demonstrations of respect and value typically occurred through interdepartmental relationships that included both social and professional interactions. Administrative inclusion facilitated connections and minimized exclusion. Cultivating program identity supported the authenticity of differing identities.

A variety of interdepartmental relationships are beneficial. Rezai M et al. also found that facilitating workplace inclusion is critical at all levels; however, the decisions of those at the top have a significant potential to impact inclusivity in the workplace [14], supporting our findings of the disproportionate impact of leadership.

The finding of administrative inclusion showed that meetings, shared spaces, and structured communication can improve successful integration. This is also consistent with literature on the impact of proximity on collaboration [15]. The final theme revealed during the focus groups was collective program identity. Despite desiring integration into the categorical programs, maintenance of a Med-Peds identity was valued for facilitating authenticity and advancing diversity. Baumeister RF et al. demonstrated that this desire for differentiation within a group is beneficial to the group as a whole. He found that groups often flourish when differences among group members are recognized and affirmed [16]. This mitigates tension between individual, subgroup, and group identities [17]. For Med-Peds and categorical residents, there is knowledge and experience to be gained from the different training.

Hwang J and Hopkins KM demonstrated that inclusion contributes to increased levels of organizational commitment and job satisfaction [18]. This supports our findings that lapses in inclusion are negatively impactful. It also highlights inclusion as a valuable goal for improving well-being.

While Med-Peds programs represent the largest cohort of combined residency programs, with 398 residents, there are 14 different combined programs, including medicine-psychiatry, medicine-dermatology, and pediatrics-emergency medicine [1]. Many of these programs are quite small, such as emergency medicine-anesthesiology, with only one resident each year, or medicine-medical genetics, with only four programs taking five residents a year. Together, however, there are 578 residents matching annually in all these combined training programs [1], which is more than many single-specialty programs, such as otolaryngology (394), neurosurgery (268), or primary care medicine programs (411) [1]. Additionally, many residents spend part of their training “off-service” rotating through different specialties; these themes of integration likely apply to such experiences as well. Thus, this project has important relevance to graduate medical education.

There are three important limitations of our study. First, despite broad participation and correlation of the findings with the national survey, the focus groups may have stifled dissenting voices or divergent opinions. A desire for conformity may have overemphasized dominant ideas and underdeveloped minority opinions. The small focus groups, with 3-5 participants, may have allowed all voices to be heard but may have limited the diversity of perspectives in each conversation. Since 33/79 (42%) of programs were represented in our convenience sample, with strong concordance of opinions, the major themes have been captured with nuanced detail.

Second, only Med-Peds program leadership was included in this study. An important next step for this project is to replicate this method among residents from a broad representation of Med-Peds programs. Similarly, replication of the study with participants from another combined specialty would support the generalizability of our findings to other combined programs.

Third, this study only considers the perspectives of program leaders in attendance at MPPDA. While a large percentage of programs attend (unpublished statistics), key voices are likely missed among those who do not attend.

## Conclusions

Successful integration of Med-Peds programs with their categorical programs has the potential to strengthen all residency programs. Survey results found that Med-Peds programs are generally well integrated. There are opportunities for improvement and common patterns that help. Exploration of what integration means and how it occurs led to findings that overlap with the literature on workplace inclusion. Findings suggest that collaborative program leadership, problem-solving, and communication are key features of integration. Similarly, a Med-Peds program that is not well integrated may be a signal of broader departmental challenges. We believe that these themes have broad applicability across academic medical centers and may serve to guide improvement, not only in Med-Peds programs but also in combined residency programs and projects that require interdisciplinary collaboration.

## Appendices

Appendix A

Interview Guide for Semi-Structured Interviews

What factors are associated with your program being integrated or not integrated?

What would be helpful to improve the integration of your program?

What factors are not helpful in the integration of the program?

Is there anything else that you can share about the integration of your program?
